# Associations between existing and newly diagnosed chronic health conditions and change in subjective life expectancy: Results from a panel study

**DOI:** 10.1016/j.ssmph.2022.101271

**Published:** 2022-10-23

**Authors:** Anushiya Vanajan, Catalin Gherdan

**Affiliations:** a Netherlands Interdisciplinary Demographic Institute, Lange Houtstraat 19, 2511 CV, The Hague, the Netherlands; b University of Groningen, University Medical Center Groningen, Department of Health Sciences, Community & Occupational Medicine, Broerstraat 5, 9712 CP, Groningen, the Netherlands

**Keywords:** Diagnosis time, Chronic diseases, Subjective longevity, Survival probability

## Abstract

**Background:**

Subjective life expectancy (SLE) is a vital predictor of mortality, health and retirement. Nevertheless, we have sparse knowledge about what drives changes in SLE. Having a chronic health condition (CHC) is probably associated with a change SLE. However, how CHCs are associated with changes in SLE may depend on whether the CHC was newly diagnosed and the type of CHC.

**Aim:**

We hypothesize that newly diagnosed CHCs will be strongly negatively associated with changes in SLE than existing CHCs. As CHCs vary in their presentation and prognosis, we differentiate associations between five CHCs - arthritis, cardiovascular diseases, sleep disorders, psychological disorders and life-threatening conditions - and changes in SLE.

**Method:**

Data from two waves of a Dutch pension panel survey, collected 3 years apart in 2015 and 2018, were used. The analytical sample included 4824 older workers between the ages of 60–65 years at wave 1. Data were analysed longitudinally using a conditional change ordered logistic regression model.

**Results:**

In general, newly diagnosed CHCs were strongly negatively associated with changes in SLE, relative to having no CHCs. Existing CHCs were also negatively associated with changes in SLE, but to a weaker strength. Interestingly, associations between CHCs and the change in SLE differed based on the CHC in question.

**Conclusion:**

Newly diagnosed life-threatening conditions, psychological disorders and cardiovascular diseases are strongly negatively associated with changes in SLE. These results provide insight into the differences in how older workers with CHCs experience late career work and how these experiences influence their SLE.

## Highlights

• SLE is a vital predictor of mortality, health and retirement timing.
• Newly diagnosed CHCs are **strongly negatively associated with changes in SLE.**
• Existing CHCs are **negatively** associated (to a lesser extent) with **changes** in SLE.
• Associations between CHCs and changes in SLE differed based on the CHC in question.
• We use panel data from Dutch older workers for whom retirement age has increased.

## 1 Introduction

To combat labour gaps that may arise from population ageing, workers across the western world are required to work up to a higher age before they can transition into retirement. However, some older workers may choose to retire early due to health-related reasons. While one is more likely to point at health-related work limitations or low vitality as drivers of early retirement preferences, recent research has begun to examine the possibility of subjective life expectancy as a driver of retirement preferences (Vanajan, Bültmann, & Henkens, 2020). Subjective life expectancy (SLE) is defined as the subjective perception of how long an individual expects to live. Older workers' assessment of how long they will live have been associated with how long they will work, when they will retire and when they will claim their pensions (Hurd, Smith, & Zissimopoulos, 2004; Khan, Rutledge, & Wu, 2014), with studies showing older workers with longer time horizons preferring later retirement (van Solinge & Henkens, 2010). Not only does SLE directly predict retirement behaviour, it also mediates the effect of poor health on older workers' early retirement preferences and their uptake of disability pension (Holman, 2019; Vanajan et al., 2020). Despite the negative consequences that low SLE may have on older workers' wellbeing and work, we do not know much about which factors or situations cause SLE to change over time. This is especially true for older working populations. Therefore, it is vital that we gather insights into what changes SLE using longitudinal methods, so as to better sustain working lives until retirement age.

Poor health has been associated with low SLE (Bae, Kim, & Lee, 2017; Liu, Tsou, & Hammitt, 2007). This is especially the case for older workers who experience chronic health conditions (CHCs). CHCs, such as depression (Kim & Lee, 2019), hypertension (Zacher, Wang, & Short, 2022) and cardiovascular diseases (Vanajan et al., 2020) have been associated with low SLE among older workers. Theoretically, these results are supported by Leventhal's Self-Regulation Theory (Leventhal, Meyer, & Nerenz, 1980). This theory states that an individual's beliefs and expectations about an illness will determine how he/she will evaluate his/her overall health situation. Older workers with CHCs may evaluate their SLE based on their beliefs about their specific CHC - its cause, its effects, its timeline and trajectory, its treatment and prognosis and/or how long the older worker has successfully managed it. Leventhal's theory demonstrates how the many characteristics of a CHC may simultaneously influence older workers' perceptions about their current and future health and their life expectancies. This theory emphasizes the importance of studying the heterogeneities in CHCs, especially in connection to SLE, which has been neglected in literature thus far.

This study examines two sources of heterogeneity in the associations between CHCs and change in SLE: whether the CHC was newly diagnosed and the type of CHC. Individuals who have been living with CHCs for a longer time may experience life differently than those who have been recently diagnosed with CHCs. On the one hand, CHCs can increase in severity with time, reaping negative consequences for SLE. For example, symptoms of arthritis have been shown to worsen with time, thereby causing an increase in activity limitations and in detrimental consequences for work and life (McDonough & Jette, 2010). On the other hand, those who have been diagnosed with a CHC earlier in life might have had the time and space required to accept, learn about and manage the needs and limitations of their condition, which could change SLE for the better. Those recently diagnosed with a CHC could still be learning about their condition, while adjusting to the functional limitations of their CHCs and finding ways to manage their CHCs in daily life (Lacaille, White, Backman, & Gignac, 2007). They could be battling the side effects of their newly prescribed medication and creating a new support system (Lacaille et al., 2007). This has been supported by a study that demonstrated the recent diagnosis arthritis to decrease functioning more than arthritis that has been diagnosed early in life (Björk, Thyberg, Haglund, & Skogh, 2006; Mutambudzi & Henkens, 2021). Despite these conflicting presumptions, to the best of our knowledge, no available studies have longitudinally examined how experiencing a CHC over a longer time period or being newly diagnosed with a CHC could be differentially associated with the change in SLE. Therefore, this study aims to investigate how existing (*existing CHCs* - diagnosed over 3 years ago) and newly diagnosed (*newly diagnosed CHCs* - diagnosed within the last 3 years) CHCs are associated with changes in SLE among older workers (60–65 years old at baseline) in the Netherlands using two waves of unique panel data. We hypothesize that being newly diagnosed with a CHC will have a stronger negative association with the change in SLE than existing CHCs: newly diagnosed *CHCs-SLE hypothesis*.

Another source of heterogeneity in the associations between CHCs and change in SLE stems from the type of CHC. CHCs differ in how they present themselves. The symptoms they pose on the human body and the intensity of these symptoms vary depending on the CHC, its severity, when it is diagnosed and how it is managed. Based on these factors, they also evoke a range of different functional limitations and demand the fulfilment of diverse needs. This necessitates the study of the associations of various CHCs separately and not as a collective group. In order to fully understand the heterogeneities in the relationship between CHCs and changes in SLE, we aim to examine how five categories of CHCs - arthritis, cardiovascular diseases, sleep disorders, psychological disorders and life-threatening diseases - are associated with changes in SLE among older workers. We chose to study these categories of CHCs because they are the most prevalent and most burdensome among older adults (OECD/European Union, 2018; WHO, 2018). They were also the most prevalent CHCs among the older workers in our sample, which was important for statistical power.

Moreover, there is a possibility that mentally-disabling CHCs mediate the associations between physically disabling conditions and the change in SLE. Physically-disabling CHCs, such as arthritis, cardiovascular disorders and life-threatening conditions, are known to be associated with sleeping problems and depleted mental health (Dickens, McGowan, Clark-Carter, & Creed, 2002; Hanssen, Nordrehaug, Eide, Bjelland, & Rokne, 2009; Sateia & Lang, 2008). We hypothesize that these negative consequences on mental health and wellbeing may, in turn, be associated with changes in SLE: *mediation hypothesis*.

This study contributes to the literature in three ways. First, this study is, to our knowledge, the first to longitudinally analyse the change in SLE using 3-year panel data from older workers. Previous studies have mostly examined SLE cross-sectionally at a single time point. The few studies that do evaluate changes in SLE, do so in a population of workers of a wider age category, patient populations or among older community dwelling adults (Chen, Zhu, Hu, & Gao, 2021; Liu et al., 2007). We study changes in SLE among 4824 older workers in pre-retirement age (between 60 and 65 years), who are burdened not only by the requirement to work longer, but also by CHCs, the prevalence of which increases with age. Presumably, this sample of older adults is at a greater risk of low SLE. However, this has not been examined longitudinally. Secondly, we study heterogeneities in the associations between CHCs and older workers' SLE. We examined two sources of heterogeneity: heterogeneity due to the type of CHC and heterogeneity due to whether CHCs were existing or newly diagnosed. This, to our knowledge, has not been previously attempted. Third, by distinguishing the associations of the short- and the long-term presentation of five categories of CHCs, we are able to identify the groups of older workers whose SLE is most affected by their CHC. Currently, literature has provided evidence on the associations between low SLE and functional limitations (Keyes & Westerhof, 2012; Kim & Lee, 2019), poor health (Bodner & Bergman, 2016), high levels of stress (Griffin, Loh, & Hesketh, 2013) and negative health practices (Scott-Sheldon, Carey, Vanable, & Senn, 2010). Based on this evidence, the groups of older workers identified by this study to have low SLE may also suffer from these additional negative effects on health and wellbeing: this may provide insights for practice efforts.

### 1.1 The retirement context in the Netherlands

Between 1960 and 2000, older and disabled workers in the Netherlands used to have easier access to generous early work exit schemes such as early retirement and disability insurance. As the effects of population ageing became more prominent, the Dutch government restricted access to early work exit schemes. For example, in 2012, the Netherlands discontinued all early retirement schemes. While early retirement is still an available option for Dutch workers, it is financially disadvantageous and is only used by older workers who can afford it. Similarly, disability insurance which used to cover all work- or non-work-related medical impairments even before a disability assessment was conducted, has been reformed to include stricter assessments of disability, while also holding employers responsible for disability wages (in the first two years of sick leave) and reintegration of disabled workers (Koning & Lindeboom, 2015).

Moreover, the Dutch government also increased the public pension age. Current retirement policies link every projected year of increase in life expectancy with an increase in public pension eligibility age by 8 months. In line with current life expectancy projections, statutory retirement age for those born in 1970 will be 68 years, while statutory retirement age for those born in 1990 will increase up to 69 years and 6 months (Oude Mulders, Henkens, & van Dalen, 2021).

The many measures taken to retain a sufficient labour force have led to extended working lives for *all* workers (Oude Mulders, 2019). In the Netherlands, the nett labour participation of older workers between 60 and 65 years rose from 21.7% in 2003 to 62.8% in 2020 (Centraal Bureau voor de Statistiek, 2021). This extension of working years occurs regardless of the heterogeneities in older workers' capacities to work and despite the health challenges that older workers may experience. Given that the prevalence and burden of CHCs increases with age, older Dutch workers may also experience more CHC-related needs and limitations at work.

## 2 Methods

### 2.1 Population

This study used data from the first and second waves of the NIDI Pension Panel Study (NPPS) that were conducted in 2015 and 2018 in the Netherlands. The NPPS is a prospective cohort study that follows a large sample of employed older workers aged between 60 and 65 years at wave 1 (Henkens, Van Solinge, Damman, & Dingemans, 2017).

Older workers were recruited using a stratified sampling approach. First, a sample of organizations were selected from the files of three large pension funds in the Netherlands (namely, ABP, PfZw and BpBouw) along the dimensions of the organization's size and sector. Together these pension funds represent 49% of wage employed workers in the Netherlands (De Nederlandsche Bank, 2015). Second, 15,470 older workers aged between 60 and 65 years who worked at least 12 h per week were randomly sampled from within the selected organizations. They received the NPPS questionnaire by post. A total of 6793 older workers responded to this questionnaire at wave 1, corroborating to a net response rate of 44%.

Between the two waves 98 respondents dropped out due to attrition (of them, 86 were deceased). A total of 6695 questionnaires were sent out by the second wave, of which 5312 were completed and returned (a net response rate of 79.3%). From this number we excluded 340 respondents who completed a shorter version of the questionnaire that did not include all relevant variables. Additionally, 147 respondents who did not respond to the item on subjective life expectancy at wave 2 and 1 respondent who had a missing value in which organization he/she belonged to were excluded from our sample. We did encounter missing values in the remaining responses. However, these missing values accounted for less than 5% of all observations. This allowed for the use of less vigorous missing data imputation methods (Little, Jorgensen, Lang, & Moore, 2014). Thus, missing data, except those of the dependant variables, were imputed using single stochastic regression imputation (Enders, 2010). Our final sample consisted of 4824 older workers (N = 4824) between the ages of 60–65 years at wave 1.

### 2.2 Measurements

#### 2.2.1 Outcome variable

Subjective life expectancy at both waves was assessed using a single item measure which inquired “How likely are you to live beyond the age of 80?” with response categories originally ranging from *highly likely* (1) to *highly unlikely* (5) on a five-point Likert scale. The responses were reverse coded to range from *highly unlikely* (1) to *highly likely* (5), with higher values representing higher subjective life expectancy. Based on this method, two categorical measures of subjective life expectancy were created at wave 1 (SLE at wave 1, five categories) and wave 2 (SLE at wave 2, five categories).

#### 2.2.2 Primary explanatory variables

The Limiting Longstanding Illness (LLSI) measure (Bajekal, Harris, Breman, & Woodfield, 2004) was used to measure the existence and new diagnosis of five categories of CHCs: arthritis, cardiovascular diseases, sleep disorders, psychological disorders and life-threatening conditions. Respondents were asked “Do you have one or more of the following longstanding diseases (as diagnosed by a doctor)" at both waves. The LLSI was followed by a list of CHCs. Respondents answered by indicating whether they were diagnosed with the particular CHC. Based on responses to the LLSI at *wave 1*, we created five dichotomized variables that represent existing CHCs (*1 = I have this CHC, 0 = I do not have this CHC*). Responses to both waves of the LLSI were used to create five dichotomized variables that represent newly diagnosed CHCs. The new diagnosis of a specific CHC was coded 1 if respondents affirmed, they were diagnosed with the CHC at wave 2 in the absence of an affirmative diagnosis of the same CHC at wave 1 (*1 = I have been newly diagnosed with this CHC, 0 = I have had this CHC for the last 3 or more years or I do not have this CHC*).

#### 2.2.3 Covariates

In order to better estimate the associations between existing and newly diagnosed CHCs and the change in SLE, we controlled for several demographic, health-related, interpersonal and work-related variables. The prevalence of CHCs increases with age and studies have shown sex differences in the prevalence, severity and patterns of CHCs among the older population (Abad-Díez et al., 2014; de Breij, Huisman, Boot, & Deeg, 2022). Moreover, past studies have shown that lower educated older workers are more likely to have health problems than higher educated older workers (de Breij et al., 2020). Socioeconomic differences among older workers have also been shown to strongly predict life expectancy (Lallo & Raitano, 2018). Education level, especially, has strongly been associated with subjective life expectancy (van Solinge & Henkens, 2018). Based on this literature, we controlled for age, sex and education level at wave 1 within the cluster of demographic variables. Age was treated as a continuous variable, measured in years. Sex was dichotomized, with 1 representing males. In the questionnaire education was originally categorized as elementary school (1), lower vocational education (2), lower general secondary education (3), intermediate vocational education (4), upper general secondary education (5), higher vocational education (6) and university education (7). For this article, we treated education as a categorical measure with 3 categories: low (includes the original categories - elementary school, lower vocational education and lower general secondary education), moderate (includes the original categories - intermediate vocational education and upper general secondary education) and high education (includes the original categories - higher vocational education and university education).

Individuals with higher levels of social support are expected to live longer (Ross & Mirowsky, 2002): this is especially true for individuals who have experienced physical health impairments. Social support is also associated with higher quality of life among older adults (Fernández–Ballesteros, 2002). Based on these results, we controlled for social support under the category of interpersonal control variables. Social support, derived from wave 1 data, was dichotomized and coded 1 if respondents affirmed that they had plenty of people they could lean on when they had problems.

Older workers with comorbid CHCs were more likely to exit paid employment compared to older workers without comorbidities (Oude Hengel, Robroek, Eekhout, van Der Beek, & Burdorf, 2019). Therefore, within the group of health-related controls, we adjusted for multimorbidity at wave 1. Multimorbidity was constructed as a dichotomized variable which was coded 1 if respondents confirmed that they were diagnosed with two or more CHCs (1 = diagnosed with two or more CHCs).

Moreover, we controlled for two work-related variables: manual work and employment status. Manual work has been associated with lower vitality, higher physical job demands and higher physical health impairments (Mutambudzi & Henkens, 2021; Schaufeli & Taris, 2014; Vanajan, Bültmann, & Henkens, 2021). Past research has also shown differences in healthy life expectancy between older adults in high and low occupational positions (Head et al., 2019). These studies made it necessary to control for job type, i.e., whether older workers were in manual or non-manual work, in our analyses. Manual work was coded 1 if respondents' jobs were associated with manual work according to the International Standard Classification of Occupation at wave 1 (Ganzeboom, 2010). Moreover, having a CHC has been associated with early retirement preferences among older workers (Vanajan et al., 2020). Retirement may provide more time for rest, recovery and leisure without the demands of work. Because employment status can influence older workers' ability to effectively manage their CHC, we controlled for employment status in our analyses. Employment status was coded 1 if respondents indicated that they do work for pay in response to the question “Which situation applied to you?” at wave 2. More details on the wording of survey questions and the coding and psychometric properties of variables are presented in Supplementary Table 1.

### 2.3 Analyses

We used conditional change ordered logistic regression analysis to longitudinally study the associations between five existing and five newly diagnosed CHCs and changes in SLE. Based on guidelines from Aickin (2009), we regressed the dependent variable at wave 2 (SLE at wave 2) on the dependent variable at wave 1 (SLE at wave 1), independent variables (existing and newly diagnosed CHCs) and control variables (demographic, health-related, interpersonal and work-related controls) (Aickin, 2009). Regressing the dependent variable at wave 2 against its baseline measure and all other covariates is mathematically equivalent to regressing the change of the dependant variable against its baseline measure and all other variables. Because of this mathematical equivalency, the coefficients resulting from the conditional change analysis can be interpreted as change effects from wave 1 to wave 2. Moreover, the inclusion of the dependent variable at wave 1 as a covariate, controls for a possible ceiling effect. Because our data is multilevel in structure (older workers were nested within organizations), we used robust standard errors clustered based on organizational belonging. The regression equation for the model derived through the conditional change ordered logistic regression analysis is presented in the supplementary file.

#### 2.3.1 Mediation analysis

In order to formerly test how mentally-disabling conditions may mediate the associations between physically-disabling CHCs and change in SLE, we conducted two Karlson, Holm and Breen (KHB) mediation analyses (Stata 14: khb): one for existing CHCs and the other for newly diagnosed CHCs. The KHB method provides unbiased decompositions of total effects into direct and indirect effects for both linear and nonlinear models (Breen, Karlson, & Holm, 2013). Within this study, the direct effect examines the association between physically-disabling CHCs and SLE, while indirect effects explore mediation by mentally-disabling CHCs.

## 3 Results

### 3.1 Descriptive statistics on key variables

**Fig. 1:**
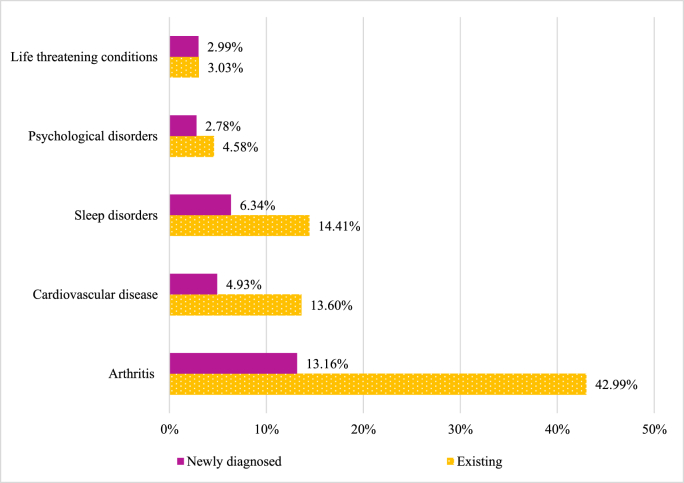
Percentage of older workers with existing CHCs and newly diagnosed CHCs.

The proportions of older workers with existing and newly diagnosed CHCs are demonstrated in Fig. 1. By the first wave, 43.5% of older workers reported having arthritis, 13.7% reported having a cardiovascular disease, 14.6% reported experiencing a sleep disorder, 4.7% experienced a psychological disorder and 3.1% reported having a life-threatening condition. Between the two waves, 12.7% of older workers were newly diagnosed with arthritis, while 4.9% were newly diagnosed with a cardiovascular disease, 6.3% reported a newly diagnosed sleep disorder, 2.8% newly experienced a psychological disorder and 2.9% were newly diagnosed with a life-threatening condition.

Older workers also experienced changes to their SLE between the two waves. The extent of these changes, however, depended on whether older workers experienced no CHCs, existing CHCs or newly diagnosed CHCs. At wave 1, the mean SLE of older workers with newly diagnosed CHCs was 3.36. This number increased slightly to 3.40 by wave 2. Older workers with existing CHCs also experienced an increase in mean SLE from 3.30 to 3.45 between the two waves. A similar increase in mean SLE was seen for older workers who did not have one of the five CHCs by wave 2. At wave 1, older workers with no CHCs had a mean SLE of 3.60, which increased to 3.78 by wave 2.

### 3.2 Results of the conditional change ordered logistic regression analysis

Table 1 demonstrates the associations between existing and newly diagnosed CHCs and the change in SLE from wave 1 to wave 2.

**Table 1:** The associations between existing and newly diagnosed arthritis, cardiovascular disease, sleep disorders, psychological disorders and life-threatening conditions and the change in subjective life expectancy from wave 1 to wave 2 (N = 4824).

Predictors	Change in subjective life expectancy from w1 to w2	
	Coef.	SE
Subjective life expectancy at w1	1.84**	0.05

*Existing*		
Arthritis	0.10	0.11
Cardiovascular disease	−0.39**	0.09
Sleep disorders	−0.19*	0.09
Psychological disorders	0.03	0.14
Life-threatening conditions	−0.18	0.18

*Newly diagnosed*		
Arthritis	−0.14	0.09
Cardiovascular disease	−0.48*	0.14
Sleep disorders	−0.29*	0.14
Psychological disorders	−0.54*	0.20
Life-threatening conditions	−1.44**	0.27

*Demographic control variables*		
Age	0.04*	0.02
Sex (reference = male)	−0.05	0.06
Education	0.10*	0.04

*Interpersonal control variables*		
Social support (reference = has social support)	0.20	0.18

*Health-related control variables*		
Multimorbidity (reference = diagnosed with two or more CHCs)	−0.26*	0.12

*Work-related controls*		
Employment status (reference = employed)	−0.06	0.06
Manual work (reference = manual worker)	−0.24*	0.09

Wald chi2 (26)	1605.85**	
Log pseudolikelihood	−4553.68	
Pseudo R^2^	0.22	

*Note.* *p < 0.05,**p < 0.001, Coef. = coefficient; SE = robust (clustered) standard error; w1 = wave 1; w2 = wave 2.

#### 3.2.1 Existing CHCs

The results revealed existing cardiovascular diseases (b = −0.39, p < 0.001) and existing sleep disorders (b = −0.19, p < 0.05) to be moderately negatively associated with changes in SLE between wave 1 and wave 2, relative to those with no CHCs**.** This association was not evident for existing arthritis, existing psychological disorders or existing life-threatening conditions.

#### 3.2.2 Newly diagnosed CHCs

Almost all newly diagnosed CHCs were negatively associated with changes in SLE compared to having no CHCs; this (partially) confirms our newly diagnosed CHCs-SLE hypothesis. Newly diagnosed life-threatening conditions saw the strongest negative association with the change in SLE (b = −1.44, p < 0.001). Newly diagnosed psychological disorders (b = −0.54, p < 0.05), cardiovascular diseases (b = −0.48, p < 0.05) and sleep disorders (b = −0.29, p < 0.05) were moderately negatively associated with changes in SLE. This association was not evident for older workers who were newly diagnosed with arthritis between the two waves.

### 3.3 Results of the mediation analysis

The results of the KHB mediation analyses are presented in Supplementary Tables 2 and 3 According to the results, almost all existing and newly diagnosed physically-disabling CHCs were directly associated with SLE at wave 2. The only exception to this pattern was existing arthritis, as the association between existing arthritis and older workers' SLE at wave 2 was mediated to a large extent (around 85%) by existing sleep disorders. Thus, our *mediation hypothesis* was only supported for existing arthritis.

## 4 Discussion

The increase in retirement age is requiring older workers in the Netherlands to work longer while experiencing existing or newly diagnosed CHCs. These older workers may be at a greater risk of low SLE, which has been shown to have negative consequences on older workers' well-being and work. Therefore, it is important to get a better understanding of how the heterogeneous experiences of CHCs might influence older workers' perceptions of their longevity in the latter years of their careers. This study, to the best of our knowledge, is the first to differentiate the associations between five existing and newly diagnosed CHCs and changes in SLE among older workers in pre-retirement age using panel data. According to descriptive statistics, generally, older workers experienced an increase in SLE between the two waves. As older workers survive up to later ages, they may become more optimistic about their longevity (Frederick & Loewenstein, 1999). They may also better adapt to the limitations of their CHCs. However, how much SLE increased differed based on whether older workers experienced no, existing or newly diagnosed CHC, with those newly diagnosed with CHCs experiencing the smallest increase in SLE between the two waves. These descriptive results corroborate with the findings from our longitudinal analysis: while some existing CHCs were negatively associated with changes in SLE, newly diagnosed CHCs were more frequently and strongly negatively associated with changes in SLE. Taken together, the results of the descriptive and regression analysis showed that SLE does not decrease over time for older workers with existing, newly diagnosed or no CHCs. Instead, SLE increases over time: but this increase was much smaller for older workers with newly diagnosed CHCs than older workers with no or existing CHCs. Moreover, this study found the extent to which CHCs changed SLE depended on the CHC in question. Thereby, our findings provide partial support to our newly diagnosed CHCs-SLE hypothesis.

### 4.1 Heterogeneities due to whether the CHC was existing or newly diagnosed

#### 4.1.1 Existing CHCs

According to the findings of our longitudinal analysis existing cardiovascular diseases and sleep disorders were negatively associated with changes in SLE. This finding is also supported by that of the mediation analysis, which shows existing cardiovascular diseases to have a direct association with SLE at wave 2. Interestingly, existing sleep disorders mediated the association between existing arthritis and SLE at wave 2.

The severity of cardiovascular diseases and sleep disorders could worsen over time or with ageing, reaping negative consequences for SLE. The worsening of these conditions might lead to adverse health events, such as strokes, myocardial infarctions or sleep attacks, the fear or expectation of which may in turn influence how older workers evaluate their life expectancy. The effect of these conditions, especially sleep disorders, could be cumulative and delayed: the longer one suffers from sleep disorders, the greater the toll its symptoms will have (Altevogt & Colten, 2006). Based on this, it may take time for the consequences of sleep disorders to add up and influence SLE.

Moreover, these results also show that, regardless of how long older workers were managing their CHCs, their SLE was still affected by the mere fact that they had a CHC. Leventhal's Self-Regulation Theory (Leventhal et al., 1980) explains that individuals build their beliefs and expectations about their overall health, wellbeing and by extension their SLE on the severity, prognosis and timeline of their illness and the consequences it has on their lives. Older workers who have been managing their CHCs for a long time, especially those whose CHCs are increasing in severity, may be more negative about their prognosis and the possibility of an illness-free life, which might be reflected in their SLE.

#### 4.1.2 Newly diagnosed CHCs

When it comes to newly diagnosed CHCs, longitudinal findings showed that all, except newly diagnosed arthritis, were negatively associated with changes in SLE (relative to having no CHCs). This (partially) supported our newly diagnosed CHCs-SLE hypothesis. These results somewhat collaborate with findings from our mediation analysis which revealed direct associations between all newly diagnosed physically-disabling CHCs and SLE at wave 2.

Older workers who are newly diagnosed with a CHC might still be adjusting to the idea of being ‘sick’. They could still be learning about and adapting to the volatile nature of their symptoms and the side effects of their newly prescribed medications (Lacaille et al., 2007). Their self-efficacy in managing their disease might be poor at the beginning and they would need to newly build a system of physical and mental support to manage their CHC (Lacaille et al., 2007). At work, the experience of a newly diagnosed CHC could cause an incongruity between job demands and the ability to perform daily work duties (Gilworth et al., 2003). These negative consequences of newly diagnosed CHCs could compound to affect older workers' assessments of their longevity.

### 4.2 Heterogeneity stemming from the type of CHC

CHCs differed in the ways through which they influenced SLE. Arthritis, whether existing or newly diagnosed, was not associated with changes in SLE. This is in line with past studies that showed no association between arthritis and SLE (Vanajan et al., 2020). While arthritis limits the functional ability of individuals, it is not terminal. Due to its high prevalence, strategies to manage arthritis are widely known and easily practiced. For example, it is relatively easy to provide ergonomic workplace adaptations to individuals with arthritis, compared to interventions for psychological or sleep disorders which would need to be personalized.

On the contrary, both existing and newly diagnosed cardiovascular diseases and sleep disorders were negatively associated with changes in SLE. The negative association between newly diagnosed cardiovascular diseases/sleep disorders and change in SLE was stronger than that between existing cardiovascular diseases/sleep disorders and change in SLE. Existing sleep disorders were also a key player in the pathway between existing arthritis and SLE. This means that even though arthritis did not directly influence SLE, older workers who may experience sleep disorders as a side effect of their arthritis may experience changes to their SLE. With regards to cardiovascular diseases, our results strengthen the argument that individuals with cardiovascular diseases continue to fear for their longevity regardless of how long they have lived with the disease (Albarqouni, von Eisenhart Rothe, Ronel, Meinertz, & Ladwig, 2016; Whitehead, Strike, Perkins-Porras, & Steptoe, 2005). This could be attributed to the unpredictability and sudden morbidity of the disease. This result is supported by previous studies that have shown prevalent cardiovascular diseases to be associated with low SLE (Zacher, Wang, & Short, 2022) and to influence older workers' preference to retire early by reducing SLE (Vanajan et al., 2020). Sleep disorders are characterized by fatigue, impaired cognitive performance, anhedonia and an inability to cope with stress. Previous research has associated the experience of a sleep disorder with low vitality, low quality of life (Reimer & Flemons, 2003; Vanajan et al., 2020) and diminished work performance (Knebelmann & Prinz, 2016), all if which could reap significant consequences to SLE.

Existing psychological disorders and life-threatening conditions have no association with changes in SLE. This could be a result of adaptation; older workers with these conditions may have adapted to and found strategies to better manage the limitations of their CHCs. Moreover, the longer they survive while experiencing their CHC, the more optimistic they may become about their longevity (Frederick & Loewenstein, 1999). However, newly diagnosed psychological disorders and life-threatening conditions were strongly negatively associated with changes in SLE. Psychological disorders present a wide range of symptoms, from suicidal ideation, anhedonia and fatigue to a lack of control (American Psychiatric Association, 2013), which can contribute to low SLE (Mirowsky, 1997). Life threatening conditions are terminal by definition. Previous studies have shown associations between being diagnosed with a life-threatening condition, such as cancer, and low SLE (Chen et al., 2021; van Solinge & Henkens, 2018), thus supporting our results.

### 4.3 Strengths

This study has several noteworthy strengths. To our knowledge, it is the first to examine the heterogeneities in how CHCs are associated with changes in SLE. SLE in itself is a relatively novel and unexplored concept in health-retirement literature, even though it plays an important role in how older workers perceive, feel about and plan their present and future lives (Hurd et al., 2004; Khan et al., 2014; van Solinge & Henkens, 2010). By distinguishing the associations between five categories of existing and newly diagnosed CHCs and changes in SLE, we provide greater insights to literature and practice on the intricate ways CHCs are associated with SLE. Moreover, we studied these associations using panel data on a unique sample of older workers, aged between 60 and 65 years. The recent increase in retirement age and the restriction of early work exit routes in the Netherlands has exposed this group of older workers to many challenges at work**.** Because older workers are more likely to be diagnosed with a CHC at older ages, older workers of pre-retirement age are facing the challenge of managing the needs and limitations of their existing or newly diagnosed CHC while working into old age. Therefore, it is vital that research gains a deeper understanding of the context, causes and consequences of the issues older workers with CHCs face at work. This knowledge can also be used to provide direction for practice initiatives.

### 4.4 Limitations

#### 4.4.1 Conceptual limitations

Our study is not without its limitations. When we say our study focuses on five CHCs, we are actually studying the associations between five categories of CHCs and change in SLE. Due to the structure of the LLSI measure, we did not have information on the individual conditions in each category of CHC. Some of the CHC categories contain numerous conditions that range in the nature and severity of symptoms. This is especially true for psychological disorders, as different psychological conditions pose varying mortality risks (Walker, McGee, & Druss, 2015). Perhaps, future studies could dig deeper into the categories of CHCs to examine how different conditions within each category influence changes in SLE. Moreover, past studies have shown an association between SLE and partners' health, family longevity, personality and general optimism/pessimism (Denollet et al., 1996; van Solinge & Henkens, 2010; Zick et al., 2014; Griffin, Loh, & Hesketh). We, however, do not have access to these measures in our data, which limits our ability to test or control for these factors within our analysis.

#### 4.4.2 Limitations related to measurements

We use survey data, which comes with limitations such as recall bias and social desirability. Besides, we use a single-item measure of SLE. Future studies might consider using a psychometrically safer multi-item measure. Moreover, some of the measures used have not been validated for our sample. There are better measures for these constructs that could be methodologically safer. For example, multimorbidity can be measured using the psychometrically supported Charlson and Elixhauser comorbidity index (Simard, Sirois, & Candas, 2018), which is unavailable in our survey. Moreover, our data is derived from two waves of a panel study. It would be better to have several time points before and after the diagnosis of CHCs to evaluate how SLE changes with time. Future studies with access to longitudinal data on SLE should consider rerunning this analysis with more waves and with a life course perspective in mind. Due to the nature of our data, we are also not able to precisely quantify how long older workers with existing CHCs have been living with their CHC. Future studies could look at how having CHCs for specific time periods (for instance, 0–3 years, 3–6 years, or more than 6 years) could influence changes in SLE. Reverse causation may also be a cause for concern in our findings: better estimates of the direction of associations could be achieved using longitudinal data with more waves.

#### 4.4.3 Limitations related to the sample

While our total sample size was relatively sizable, the number of cases diagnosed with specific CHCs at the two timepoints was smaller. Moreover, the choice of our study sample brings with it two limitations. First, it limits the generalizability of our results across other age groups (e.g., younger workers with CHCs), countries (with different retirement landscapes), and between other groups of Dutch older workers who are not attached to pension funds (such as self-employed older workers because the NPPS collected data from older workers attached to pension funds). Second, for this analysis we limit our sample to older workers who are working at wave 1, which means that our analysis does not capture older workers with severe complaints/limitations who might have left the workforce before wave 1.

## 5 Conclusion

This study is the first to investigate the heterogeneities in the associations between five existing and five newly diagnosed CHCs and changes in SLE among older workers. In general, older workers with existing, newly diagnosed or no CHCs experienced an increase in their SLE with time. However, this increase was much smaller for older workers newly diagnosed CHCs than for older workers without CHCs or with existing CHCs. Moreover, how CHCs changed SLE differed based on the CHC in question, with newly diagnosed life-threatening conditions and psychological disorders being associated with the worst outcomes for older workers' SLE. Together, these findings provide us with deeper insights into how older workers with CHCs interpret their future (work) capacities and estimate their life expectancy. In order to sustain work tenures of older workers' with (especially newly diagnosed) CHCs (Vanajan et al., 2020), it is vital to ensure that these groups of older workers are better supported and well-accommodated in the last stages of their careers.

## Ethical statement

Hereby, I, Anushiya Vanajan, corresponding author of manuscript titled “Associations between existing and newly diagnosed chronic health conditions and subjective life expectancy: Results from a panel study” consciously assure that the following is fulfilled for the manuscript:

1. Participant consent for participation and publication was obtained during data collection.
2. This material is the authors' own original work, which has not been previously published elsewhere.
3. The paper is not currently being considered for publication elsewhere.
4. The paper reflects the authors' own research and analysis in a truthful and complete manner.
5. The paper properly credits the meaningful contributions of co-authors and co-researchers.
6. The results are appropriately placed in the context of prior and existing research.
7. All sources used are properly disclosed (correct citation). Literally copying of text must be indicated as such by using quotation marks and giving proper reference.
8. All authors have been personally and actively involved in substantial work leading to the paper, and will take public responsibility for its content.

## Author contributions

C.G. performed the statistical analysis and wrote the initial draft of the paper. A.V. supervised the data analysis and the writing process and developed the final draft of the paper.

## Funding

This work was supported by the Netherlands Organization for Scientific Research (grant number 453-14-001 to Prof. Kène Henkens).

## Availability of data, materials or code

The data that support the findings of this study are available from the corresponding author, A.V., upon reasonable request.

## Declaration of competing interest

We have no conflicts of interests or competing interest to declare.

## Data Availability

Data will be made available on request.
