# Supplementary material for: Associations between existing and newly diagnosed chronic health conditions and change in subjective life expectancy: Results from a panel study
Source: SSM Popul Health. 2022 Oct 23;20:101271. doi: 10.1016/j.ssmph.2022.101271 (PMC9619028; doi:10.1016/j.ssmph.2022.101271)
Supplement: Multimedia component 1 [file mmc1.docx]

**Supplementary Material**

**Supplementary Methods**

**Analysis**

The regression equation for the model derived through the conditional change ordered logistic regression analysis:

$SLE at wave 2=\beta_{0}+\beta_{1}SLE at wave 1+\beta_{2}existing arthritis+\beta_{3}existing cardiovascular diseases+\beta_{4}existing sleep disorders+\beta_{5}existing psychological disorders+\beta_{6}existing life-threathening conditions+\beta_{7}newly diagnosed arthritis+\beta_{8}newly diagnosed cardiovascular diseases+\beta_{8}newly diagnosed sleep disorders+\beta_{9}newly diagnosed psychological disorders+\beta_{10}newly diagnosed life-threathening conditions+\varepsilon$

**Additional heterogeneity analysis between older manual and non-manual workers**

Past studies have shown that life expectancy and labour participation may differ between social groups; job type, especially, is an important source of heterogeneity in life expectancy (Head et al., 2019; Lallo & Raitano, 2018). Older workers in lower occupational positions tend to hold their healthy life expectancy in poorer regard than older workers in higher occupational positions (Head et al., 2019). To test for this heterogeneity, we conducted a conditional change ordered logistic regression analysis where we tested for the interaction between manual work and existing and newly diagnosed CHCs on change in older workers’ SLE.

**Supplementary results**

**Additional heterogeneity analysis**

Results of the additional conditional change ordered logistic regression analysis, which examined interactions between manual work and CHCs on change in SLE of older workers, is presented in Table 4 of the supplementary material. According to the results, older workers in manual labour who were newly diagnosed with a psychological disorder were more likely to experience declines in SLE (*b*=-1.36, p<0.05). No other interaction effects were significant.

Table 1.

Details on the wording of survey questions, coding of variables and psychometric properties of each variable (N=4,824)

| Variables | Wording of survey question | Coding (frequencies or descriptive statistics) |
| --- | --- | --- |
|  |  |  |
| *Dependant variables* |  |  |
| Subjective life expectancy at w1 | How likely are you to live beyond the age of 80?  (5 answer categories on a Likert scale from 1=highly likely to 5=highly unlikely, which was reverse coded for analysis) | Categorical variable, ranging from 1 to 5  1=Highly unlikely (w1=2.40%, w2=1.78%)  2=Rather unlikely (w1=7.73%, w2=5.29%)  3=Middling (w1=44.47%, w2=40.13%)  4=Rather likely (w1=36.21%, w2=40.94%)  5=Highly likely (w1=9.18%, w2=11.86%) |
| Subjective life expectancy at w2 |  |  |
|  |  |  |
| *Independent variables* |  |  |
| Arthritis - Existing | Do you have one or more of the following longstanding diseases, as diagnosed by a doctor?  (answer structure: chose between yes or no) | Dichotomized variable:  1=I have this CHC (42.99%)  0=I do not have this CHC (57.01%) |
| Arthritis - Newly diagnosed |  | Dichotomized variable:  1=I have been newly diagnosed with this CHC (13.16%)  0=I have had this CHC for the last 3 or more years or I do not have this CHC (86.84%) |
| Cardiovascular diseases - Existing | Do you have one or more of the following longstanding diseases, as diagnosed by a doctor?  (answer structure: chose between yes or no) | Dichotomized variable:  1=I have this CHC (13.60%)  0=I do not have this CHC (86.40%) |
| Cardiovascular diseases - Newly diagnosed |  | Dichotomized variable:  1=I have been newly diagnosed with this CHC (4.93%)  0=I have had this CHC for the last 3 or more years or I do not have this CHC (95.07%) |
| Sleep disorders - Existing | Do you have one or more of the following longstanding diseases, as diagnosed by a doctor?  (answer structure: chose between yes or no) | Dichotomized variable:  1=I have this CHC (14.41%)  0=I do not have this CHC (85.59%) |
| Sleep disorders - Newly diagnosed |  | Dichotomized variable:  1=I have been newly diagnosed with this CHC (6.34%)  0=I have had this CHC for the last 3 or more years or I do not have this CHC (93.66%) |
| Psychological disorders - Existing | Do you have one or more of the following longstanding diseases, as diagnosed by a doctor?  (answer structure: chose between yes or no) | Dichotomized variable:  1=I have this CHC (4.58%)  0=I do not have this CHC (95.42%) |
| Psychological disorders - Newly diagnosed |  | Dichotomized variable:  1=I have been newly diagnosed with this CHC (2.78%)  0=I have had this CHC for the last 3 or more years or I do not have this CHC (97.22%) |
| Life-threatening conditions - Existing | Do you have one or more of the following longstanding diseases, as diagnosed by a doctor?  (answer structure: chose between yes or no) | Dichotomized variable:  1=I have this CHC (3.03%)  0=I do not have this CHC (96.97%) |
| Life-threatening conditions - Newly diagnosed |  | Dichotomized variable:  1=I have been newly diagnosed with this CHC (2.99%)  0=I have had this CHC for the last 3 or more years or I do not have this CHC (97.01%) |
|  |  |  |
| *Demographic control variables* |  |  |
| Age | In what year were you born? (age in years were calculated) | Continuous variable: ranging from 60 to 65 years  (Mean=62.04, *SD*=1.59) |
| Sex | Are you a man or woman?  (2 answer categories: 1=man, 2=women) | Dichotomized variable:  1=Male (54.75%)  0=Female (45.25%) |
| Education | What is the highest level of education you’ve completed?  (7 answer categories: 1=elementary school, 2=lower vocational education, 3=lower general secondary education, 4=intermediate vocational education, 5=upper general secondary education, 6=higher vocational education, 7=university) | Categorical variable: ranging from 1 to 3  1=Low (26.14%)  2=Moderate (25.77%)  3=High (48.09%) |
|  |  |  |
| *Interpersonal control variables* |  |  |
| Social support | Could you indicate whether the following statements apply to you: There are plenty of people I can lean on when I have problems?  (3 answer categories: 1=yes, 2=more or less, 3=no) | Dichotomized variable:  1=Has social support (96.89%)  0=Does not have social support (3.11%) |
|  |  |  |
| *Health-related control variable* |  |  |
| Multimorbidity | Do you have one or more of the following longstanding diseases, as diagnosed by a doctor?  (answer structure: chose between yes or no) | Dichotomized variable:  1=Yes, has comorbidities (51.60%)  0=No, no comorbidities (48.40%) |
|  |  |  |
| *Work-related control variables* |  |  |
| Employment status | Which situation applies to you?  (2 answer categories: 1=I work for pay, 2=I do not work for pay any longer) | Dichotomized variable:  1=Employed (51.04%)  0=Not employed (48.96%) |
| Manual work | Based on the International Standard Classification of Occupation, in which category could your job be grouped? | Dichotomized variable:  1=Manual work (18.97%)  0=Non-manual work (81.03%) |

*Note. SD*=standard deviation; w1=wave 1; w2=wave 2

Table 2.

Associations between existing physically-disabling chronic health conditions and older workers’ subjective life expectancy (N=4,824)

| Existing physically-disabling chronic health conditions | Total direct effect | | Total indirect effect | | Indirect effect via existing sleep disorders | Indirect effect via existing psychological disorders |
| --- | --- | --- | --- | --- | --- | --- |
|  | OR | CI | OR | CI | % | % |
| Arthritis | 1.04 | 0.84-1.29 | 1.10* | 1.04-1.17 | 82.0 | 18.0 |
| Cardiovascular diseases | 0.50** | 0.42-0.59 | 1.00 | 0.97-1.04 | 98.9 | 1.1 |
| Life-threatening condition | 0.45** | 0.32-0.64 | 1.03 | 0.99-1.07 | 86.7 | 13.3 |

*Note*. ∗*p <*0.05, ∗∗*p <*0.001. Dependent variable is older workers’ subjective life expectancy. OR, odds ratio; CI, 95% confidence interval; %, attributable percentage

Table 3.

Associations between newly diagnosed physically-disabling chronic health conditions and older workers’ subjective life expectancy (N=4,824)

| Newly diagnosed physically-disabling chronic health conditions | Total direct effect | | Total indirect effect | | Indirect effect via newly diagnosed sleep disorders | Indirect effect via newly diagnosed psychological disorders |
| --- | --- | --- | --- | --- | --- | --- |
|  | OR | CI | OR | CI | % | % |
| Arthritis | 0.81* | 0.68-0.96 | 0.98 | 0.94-1.02 | 61.5 | 38.5 |
| Cardiovascular diseases | 0.61** | 0.46-0.79 | 0.99 | 0.95-1.04 | 174.0 | -74.0 |
| Life-threatening conditions | 0.27** | 0.18-0.42 | 0.99 | 0.95-1.03 | 14.5 | 85.5 |

*Note*. ∗*p <*0.05, ∗∗*p <*0.001. Dependent variable is older workers’ subjective life expectancy. OR, odds ratio; CI, 95% confidence interval; %, attributable percentage

Table 4.

The effects of manual work and existing and newly diagnosed arthritis, cardiovascular disease, sleep disorders, psychological disorders and life-threatening conditions on change in subjective life expectancy from wave 1 to wave 2 (N = 4,824)

| Predictors | Change in subjective life expectancy  from w1 to w2 | |
| --- | --- | --- |
|  | Coef. | SE |
|  |  |  |
| Subjective life expectancy at w1 | 1.86** | 0.05 |
|  |  |  |
| *Existing CHCs * Manual work* |  |  |
| Arthritis * Manual work | 0.09 | 0.13 |
| Cardiovascular diseases * Manual work | -0.29 | 0.21 |
| Sleep disorders* Manual work | -0.31 | 0.24 |
| Psychological disorders * Manual work | -0.25 | 0.41 |
| Life-threatening conditions * Manual work | -0.22 | 0.47 |
|  |  |  |
| *Newly diagnosed CHCs * Manual work* |  |  |
| Arthritis * Manual work | -0.08 | 0.21 |
| Cardiovascular diseases * Manual work | -0.20 | 0.30 |
| Sleep disorders * Manual work | -0.54 | 0.29 |
| Psychological disorders * Manual work | -1.36* | 0.54 |
| Life-threatening conditions * Manual work | -0.59 | 0.56 |
|  |  |  |
| *Demographic control variables* |  |  |
| Age | 0.04* | 0.02 |
| Sex (reference=male) | -0.07 | 0.06 |
| Education | 0.12* | 0.04 |
|  |  |  |
| *Interpersonal control variables* |  |  |
| Social support (reference=has social support) | 0.17 | 0.18 |
|  |  |  |
| *Health-related control variables* |  |  |
| Multimorbidity (reference=diagnosed with two or more CHCs) | -0.25** | 0.06 |
|  |  |  |
| *Work-related controls* |  |  |
| Employment status (reference=employed) | -0.06 | 0.06 |
|  |  |  |
| Wald chi2 (26) | 1472.05** | |
| Log pseudolikelihood | -4602.47 | |
| Pseudo R^2^ | 0.21 | |

*Note.* *p<0.05,**p<0.001, Coef.=coefficient; SE=robust (clustered) standard error; w1=wave 1; w2=wave 2
